# Micromachined Thermal Gas Sensors—A Review

**DOI:** 10.3390/s23020681

**Published:** 2023-01-06

**Authors:** Ethan L. W. Gardner, Julian W. Gardner, Florin Udrea

**Affiliations:** 1 Department of Engineering, University of Cambridge, Cambridge CB3 0FA, UK; 2 School of Engineering, University of Warwick, Coventry CV4 7AL, UK

**Keywords:** MEMS, sensor, gas sensor, thermal conductivity, micromachined, review

## Abstract

In recent years, there has been a growing desire to monitor and control harmful substances arising from industrial processes that impact upon our health and quality of life. This has led to a large market demand for gas sensors, which are commonly based on sensors that rely upon a chemical reaction with the target analyte. In contrast, thermal conductivity detectors are physical sensors that detect gases through a change in their thermal conductivity. Thermal conductivity gas sensors offer several advantages over their chemical (reactive) counterparts that include higher reproducibility, better stability, lower cost, lower power consumption, simpler construction, faster response time, longer lifetime, wide dynamic range, and smaller footprint. It is for these reasons, despite a poor selectivity, that they are gaining renewed interest after recent developments in MEMS-based silicon sensors allowing CMOS integration and smart application within the emerging Internet of Things (IoT). This timely review focuses on the state-of-the-art in thermal conductivity sensors; it contains a general introduction, theory of operation, interface electronics, use in commercial applications, and recent research developments. In addition, both steady-state and transient methods of operation are discussed with their relative advantages and disadvantages presented. Finally, some of recent innovations in thermal conductivity gas sensors are explored.

## 1. Introduction

The last twenty years have seen increased concern over environmental pollution from potentially harmful substances that could have an effect on our health, quality of life and operation of industrial processes. This has led to a large increase in research in the area of miniature, low-cost gas sensors, which need to be able to identify or monitor toxic and flammable chemicals in different environments, and ensure the safety of people and our ecosystem. The global market value for gas sensors was estimated at 2.5 billion US dollars in 2021, with a predicted growth rate of 8.9% Compound Annual Growth Rate (CAGR) from 2022 to 2027, highlighting an area driven by commercialisation with demand only set to increase over the next decade [1].

**Figure 1:**
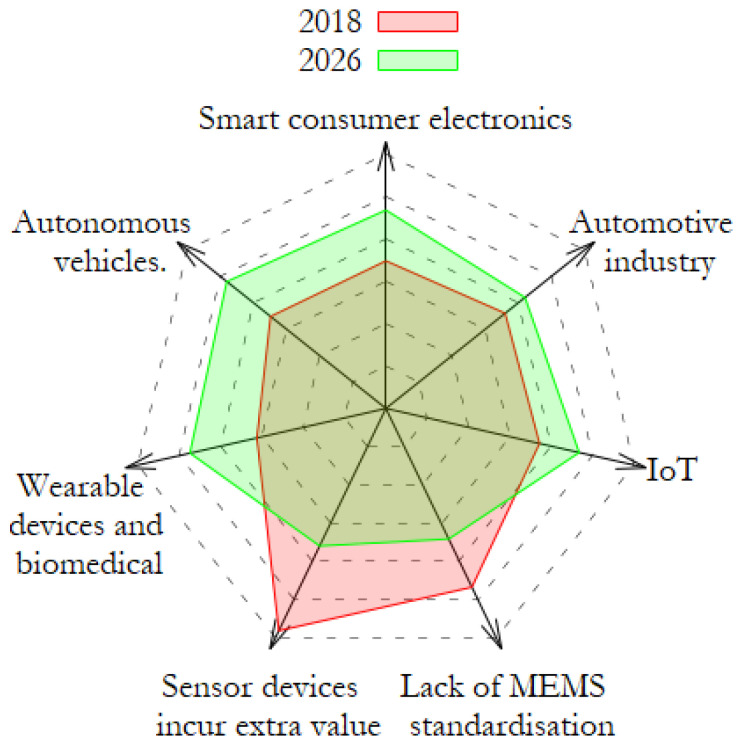
A chart showing the development of the top impacting factors on the global MEMS market from 2019 to 2026, predicted forecast data are taken from [2].

A market evaluation for MEMS by Allied Market Research [2] predicted that the largest contributing factors to the growth in MEMS sensors comes from smartphones, portable electronics, Internet of Things (IoT) and autonomous vehicles. Figure 1 graphically illustrates the predicted increasing importance of specific sectors for MEMS sensors. With the largest increases expected for wearable devices, healthcare, autonomous vehicles and smart consumer electronics. It is with the knowledge that the future landscape of MEMS is trending towards smart consumer electronics and wearable devices that sensors must become smaller, more power efficient and most importantly, smarter, i.e., use embedded Artificial Intelligence (AI).

A recent review paper on micro and nano-fabricated gas sensor technologies is given by Nazemi et al. [3]. This gives an overview on the different micromachined methods for creating gas sensors, however, it does not discuss the detection of gases using thermal phenomena. Detection of gases using gas-specific thermal conductivity has been researched for a long time, with considerable attention before the year 2000. However, in the last 5–10 years this technology has entered a renaissance period with a large resurgence of interest spanning from the research sector into the industrial sector. This resurgence can be attributed to the miniaturisation of silicon-based MEMS devices allowing dramatic improvements in power consumption, response time, manufacturing costs at volume, footprint, integration, accuracy and reliability alongside the advent of smart sensing systems and post-processing, allowing many of the drawbacks of this technology to be tackled.

The detection principle of most current gas sensors relies on chemical reactions between a gas and a chemically active material and a recent review on the most common of these technologies (metal oxide) can be found here [4]. A physical property (e.g., electrical resistance) of the chosen material is altered upon interaction with the gas. A major drawback of sensors relying on this methodology is their poor reliability, high humidity dependence, and significant drift with age, which is due to the slow poisoning/degradation of the active material. Gas sensors that depend upon only the physical properties of the target analyte avoids these significant problems.

This review focuses on micromachined gas sensors that use the physical principle of thermal conductivity. Thermal conductivity gas sensors offer several advantages that include lower cost, lower power consumption, no adsorption/catalyst, faster response time, longer lifetime and smaller footprint. However, these are coupled with the inherent drawbacks of lower sensitivity, lower selectivity and also a cross-sensitivity to ambient humidity and temperature. These are the main barriers that need to be overcome in order to develop a sensing technology that can displace current practices and develop a much larger market. If thermal gas sensors can overcome these barriers, then they have the potential to become very disruptive in the MEMS gas sensing market, with the ability of being much cheaper, smaller and lower power-consumption, all three of which are important parameters in modern day applications.

This review starts by looking at the common thermal conductivity sensing methods and the theory behind their operation, which is followed by an overview on the various methods of temperature transduction. After this introduction, a brief discussion is presented on the most common electronic interfacing. Then, commercial applications that currently use a form of this technology are reviewed followed by a discussion on the latest academic developments within the field. It is expected that the information will be useful to other working scientific and commercial groups by summating where the research is at this point in time and by helping to identify areas that future research can be directed towards.

## 2. Thermal Gas Sensing Methods

The general sensing principle behind thermal conductivity devices is based upon the same axiom that they all use the physical phenomena of heat and heat transfer to their advantage. A temperature difference exists between a hot element and a cold element, whereby there is thermal heat transfer through the investigated gas. The thermal properties of the gas will alter the heat transfer that is seen through the system, thus changing the output of the sensor. This change in output can then be related to the gas under test.

Through this description, it is evident that heat transfer to/from the investigated gas must be maximised to optimise device performance. Thus, sensor geometry, material and operating conditions must be carefully considered due to their strong influence on sensor performance. The influence of these factors is considered and highlighted throughout. This section will describe many of the operating methods that have been applied to sensors for thermal gas sensing. It is split into two main sections:

1. *Steady-State Operation*: static operation of the thermal flow sensor, meaning that the sensor biasing variables do not change with time, e.g., constant current, constant voltage, constant power and constant temperature drive modes.
2. *Transient Operation*: dynamic operation of the thermal flow sensor, meaning that the sensor biasing variables are changing with time. This includes transient hot-wire method (square wave bias), 3-omega (sine wave bias) and thermal resonance.

A summary of various methods for steady-state and transient operation are described in the following pages.

### 2.1. Steady-State Operation

#### 2.1.1. Thermal Conductivity Detector (TCD)

**Figure 2:**
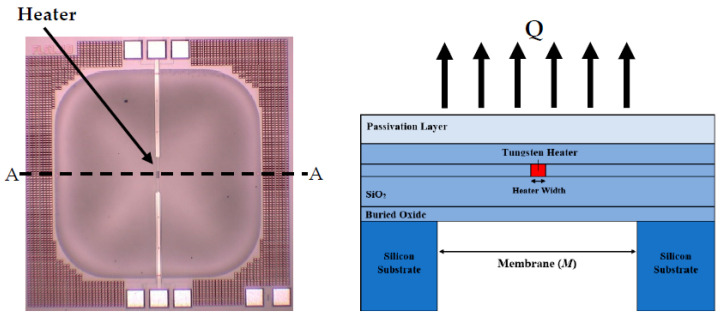
An optical micrograph of a recent thermal conductivity detector with a schematic illustration across the cut-line A-A. A micro-heater uses Joule heating to increase its temperature and heat transfer into the surrounding environment will then occur following Fourier’s Law.

The simplest thermal conductivity gas sensing method employs a direct current operation. The most basic DC technique is based upon Fourier’s Law, which states that the heat transfer through a material is proportional to both the area and the negative gradient in the temperature. This is illustrated in Figure 2 using a recently developed thermal conductivity detector as a reference [5], where $\dot{Q}$ is the heat transfer from the heater into the surrounding medium through conduction and convection.

Despite the three-dimensional geometry making it difficult to obtain an exact analytical solution of the thermal phenomena through the system, lumped system analysis can be used to describe the relationship between sensor response, gas properties and the applied power.

In steady state analysis, the energy balance implies that the ratio of heat transfer from the lumped solid object is equal to the rate of heat generation within the object, in this case the heater. Therefore, the rate of heat generation in a simplified model can be written as:

(1) $$\dot{Q}={-h}_{T}A_{s}{\left.\frac{dT_{f}}{dy}\right|}_{WALL}$$

where $\dot{Q}$ is the rate of heat generation in the heating resistor, $h_{T}$ is the heat transfer coefficient (combined for conduction and convection; n.b. radiation can be ignored as the temperatures of operation are generally too low for it to be a significant effect), $A_{s}$ is the surface area of the heated element and ${\left.\frac{dT_{f}}{dy}\right|}_{WALL}$ is the temperature gradient between the surface of the heating resistor and the ambient temperature of the gas. Substitutions can be made for the operating heater temperature, *T_H_*, and the ambient temperature of the gas, $T_{\infty }$, to give the more commonly written form of the equation:

(2) $$\dot{Q}=\frac{h_{T}A_{s}}{L}(T_{H}-T_{\infty })$$

In order to generate heat, an electrical bias is applied to a resistor and, through Joule heating, its temperature increases above ambient. The rate of heat generation is given by the dissipated electrical power, $I^{2}R$. Combining this with the known quadratic dependence of doped silicon on resistance results in the following equation:

(3) $$I^{2}R_{T0}(1+\alpha_{T}\left(T_{H}-T_{\infty }\right)+\beta_{T}{\left(T_{H}-T_{\infty }\right)}^{2})=\frac{h_{T}A_{s}}{L}(T_{H}-T_{\infty })$$

From this equation, it can be seen that the heater temperature (and therefore resistance) is related to the heat transfer from the heated resistor to the gas, which will change when a different gas is present in the atmosphere; thus, displaying the working principle of a thermal conductivity gas sensor in DC operation mode. The heat transfer coefficient, $h_{T}$, is highly dependent on the thermal conductivity of the gas. Table 1 shows the thermal conductivity values of some common gases at 300 K.

Knowing that heat transfer in the system depends on the varying thermal conductivity of the gas, it can be concluded that the detection of gases with similar values of thermal conductivity is very difficult. Common problematic examples are measuring carbon dioxide, carbon monoxide or ammonia concentration in air. This table also highlights why thermal conductivity sensing methods are often used for the detection of hydrogen and helium with much higher conductivities (see Table 1).

**Figure 3:**
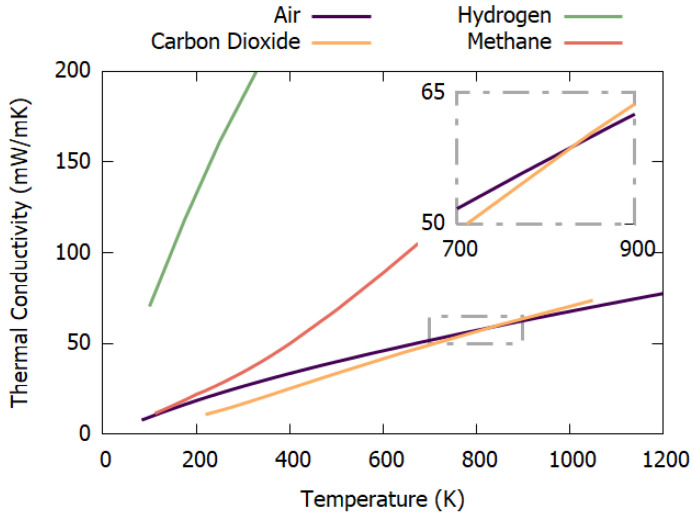
A plot showing how the thermal conductivity of air, carbon dioxide, hydrogen and methane vary with temperature. Inset—detailed view of where air and carbon dioxide intersect.

In addition to Table 1, Figure 3 graphically depicts how the thermal conductivity of air, carbon dioxide, hydrogen and methane varies with temperature. The inset highlights an area where air and carbon dioxide intersect, meaning that at this temperature (~550 °C) the thermal conductivities of the two gases are identical (~58.5 × 10^−3^ W/m·K). Due to this non-parallel evolution of thermal conductivity with temperature, an extra difficulty arises when measuring CO_2_ with heated elements. Using a hot element means the temperature of the gas around the element increases, which in turn reduces the difference in thermal conductivity between air and CO_2_ and thus decreases sensitivity. However, a higher temperature is desired in order to create a larger temperature difference with the atmosphere and induce more heat transfer. This results in a compromise that must be considered and an optimisation that must be carried out to find the ideal temperature in which to drive a heater. Figure 3 also illustrates why thermal technology is suited to the detection of hydrogen and methane in air.

**Figure 4:**
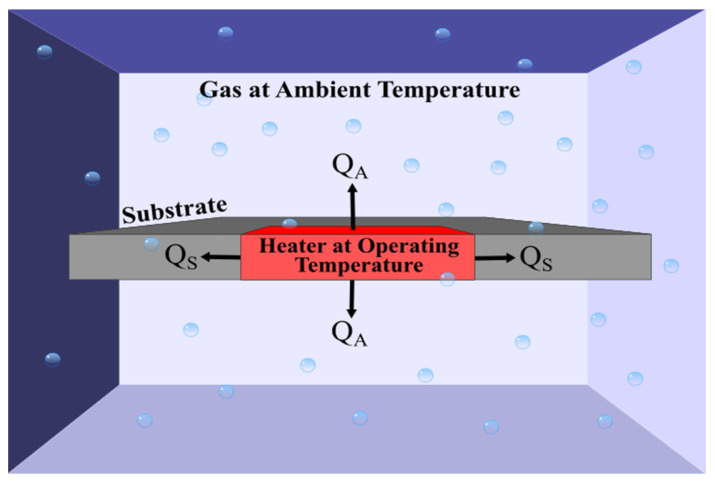
A basic illustration of the working principle behind a thermal conductivity device with the addition of a supporting structure.

Research has concentrated on improving the sensitivity of DC thermal conductivity devices to enable the accurate measurement of gases with similar lower thermal conductivities, allowing it to become comparable with other technologies, whilst providing the other inherent benefits; thus, making the technology commercially attractive. One such limiting factor of these devices is the waste heat transfer. Figure 4 shows a more realistic illustration of a thermal conductivity device where the heater is connected through a solid structure, which could be in the form of a suspended membrane, bridge or substrate. Here, a proportion of the heat transfer is going from the heater through the solid structure in the form of conduction. This is often a large proportion of the heat transfer due to solid structure have much lower thermal resistance, resulting in heat transfer through the solid structure, $Q_{S}$, being larger than heat transfer into the atmosphere, $Q_{A}$. This heat transfer is wasted because it is not interacting with the gaseous medium and thus the proportion of heater temperature change is reduced. This highlights the necessity of optimising the geometry and design of thermal conductivity devices to minimise these conduction losses, whilst also considering structural robustness and manufacturing feasibility.

#### 2.1.2. Thermal Conductivity Pellistor

Thermal conductivity pellistors (word derived from pellet + resistor) have been used for gas analysis in industry for many decades [6] with their first application being for the detection of hydrogen whilst filling airships in 1913. This is still used in industry for the analysis of binary gas mixtures containing hydrogen due to the large difference in thermal conductivity between air and hydrogen.

**Figure 5:**
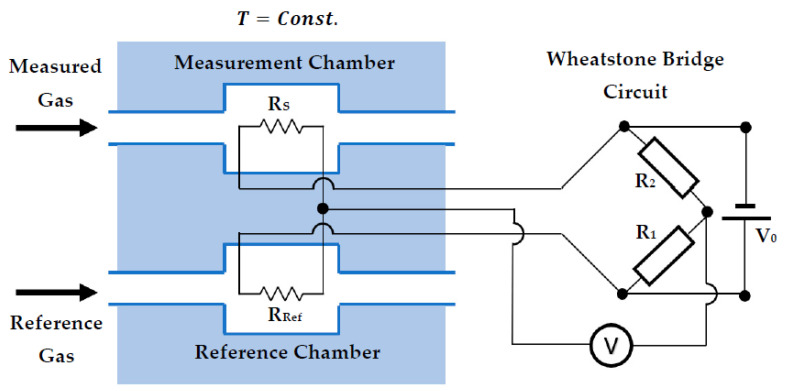
Illustration showing the working principle of a thermal conductivity pellistor with its read-out circuitry included.

The principle of operation is based upon the same mechanism outlined for TCDs, except the thermal conductivity value is dependent on the difference between two resistors; one which has been exposed to the gas under investigation and one that is exposed to a reference gas (usually air). Figure 5 is an illustration showing the working principal behind a thermal conductivity pellistor. Here, it can be seen that one resistor is exposed to the gas under investigation and the other is exposed to a reference gas. The two outputs are connected to a Wheatstone bridge which measured the resistance difference. Thus, when both resistors are exposed to the same gas, they will lose heat at the same rate which will result in the output of the bridge being zero. When the measurement resistor is exposed to a target gas of different thermal conductivity, this will exhibit a different heat loss, causing a different voltage and creating unbalance in the bridge which can be measured.

The voltage measured across a Wheatstone bridge, *V*, can be described using the following equation where $R_{S}$, $R_{Ref}$, $R_{1}$ and $R_{2}$ are resistances as shown in Figure 5 and $V_{0}$ is the voltage applied to the bridge.

(4) $$V=\left(\frac{R_{S}}{R_{S}+R_{2}}-\frac{R_{Ref}}{R_{Ref}+R_{1}}\right)V_{0}$$

It should also be noted that if $R_{1}=R_{2}\gg R_{S},R_{Ref}$ then

(5) $$V\approx \left(\frac{R_{s}-R_{Ref}}{R_{1}}\right)V_{0}$$

A more recent version of the thermal conductivity sensor holds the measurement resistor and the reference resistor at a constant temperature difference. The hot and cold resistor are positioned in the same chamber exposed to the reference gas and heat is transferred from the hot resistor to the cold resistor through the investigated gas. The power that is required to maintain the hot resistor at a constant temperature is a direct measurement of the thermal conductivity of the gas under investigation.

Thermal conductivity pellistors are often used in hydrogen applications due to its high value of thermal conductivity. One advantage of pellistors for this are their wide detection range, which spans from <1% to 100%. This is because they can operate without the presence of O_2_, unlike many other hydrogen sensor technologies. However, TC pellistors struggle to detect very low concentrations and they are often combined with an active (catalytic) pellistor in a pellistor pair. Sensor combinations of this type are common in the commercial sector due to the ability to measure both a large range and a low detection limit. However, pure TC pellistors are resistant to poisoning and offer long operating lives.

Pellistor pairs (active and passive) are also used to detect some VOCs, but only when in high concentrations that are within the region of the Lower Explosive Limit (LEL) and Upper Explosive Limit (UEL). These are already commercially available and a review of the currently commercially available sensors for the detection of VOCs, including thermal-based pellistors, can be found here [7].

### 2.2. Transient Operation

#### 2.2.1. Transient Hot Wire Method (THM)

The transient hot wire method is a very popular technique to measure the thermal conductivity of gases, liquids [8], solids [9], nanofluids [10] and refrigerants [11]. In fact, 200 years ago scientists were using an unrefined version of this method to make the first ever thermal conductivity measurements on gases. A description of the working principle behind THM is now presented. A summary of the standardised theory behind THM can be found here [12].

**Figure 6:**
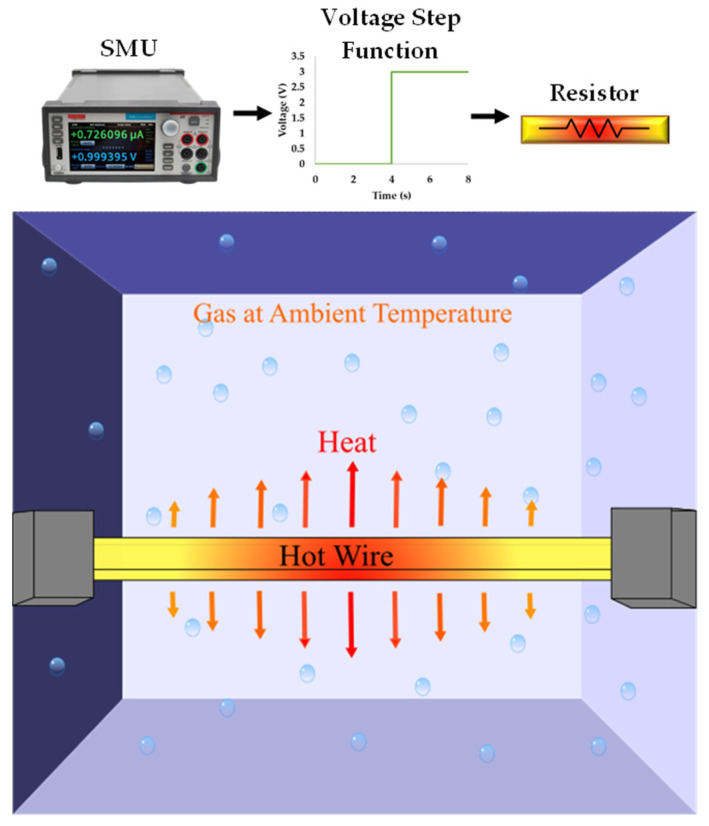
An illustration of the working principal behind the transient hot wire method. The resistor is biased with a voltage step to initiate the measurement.

In order to measure the thermal conductivity using the transient hot wire method, a voltage/current is biased across the resistor that is at thermal equilibrium within the system. The technique is based on the transient measurement of the rise in temperature seen in the resistor that is located in direct contact with the test sample. Figure 6 illustrates the working principle behind the THM. Firstly, there is an application of a step voltage across the hot wire, which in this case is provided by a Source Measure Unit (SMU). With the stepped bias voltage, the hot wire will heat up in time, due to Joule heating. This process of heating up will be different depending on the thermal conductivity of the atmospheric gas into which the hot wire can dissipate its heat.

The hot wires used in such method are very long and thin, resulting in quick heating up times (usually << 1 s). Due to these small temporal steps, there is no convection present in the measurements and thus the measurement of the thermal conductivity can be very accurate.

Equation (6) defined the rate of heat generation on the left-hand side and coupled this with heat transfer into the gas on the right-hand side, in a steady-state solution. The transient hot wire method is so temporally fast that convection can be neglected and therefore heat transfer will take place solely due to conduction, and thus can be approximated by Fourier’s heat conduction equation where $k_{f}$ is the material thermal conductivity. In addition to this, the second order term for heat generation in the hot wire can be neglected, resulting in this approximation:

(6) $$I^{2}R_{T0}(1+\alpha_{T}(T_{H}-T_{\infty }))\approx k_{f}A_{s}\left(\frac{T_{H}-T_{\infty }}{L}\right)$$

This equation can be rearranged to describe the temperature difference in the system:

(7) $$T_{H}-T_{\infty }\approx \frac{I^{2}R_{T0}}{\alpha_{T}I^{2}R_{T0}+\frac{k_{f}A_{s}}{L}}$$

From Equation (7), it can be seen that temperature change within the hot wire is inversely proportional to the thermal conductivity of the surrounding gas. This system in its transient state would result in the hot wire rising from *T_∞_* to *T_H_* over a certain period of time. Therefore, we need to add a transient term to the system. This term is derived from the heat equation and describes how the distribution of heat evolves over time. The change in temperature can be simplified for the lumped system analysis as follows:

(8) $$\frac{dT}{dt}=\alpha \nabla^{2}T=\alpha \left[\frac{\partial^{2}T}{\partial x^{2}}+\frac{\partial^{2}T}{\partial y^{2}}+\frac{\partial^{2}T}{\partial z^{2}}\right]$$

where $\nabla^{2}$ denotes the Laplace operator and α is the thermal diffusivity that can be described as a function of thermal conductivity, k, specific heat capacity at constant pressure, $C_{P}$, and density, ρ.

(9) $$\alpha =\frac{k}{C_{P}\rho }$$

Adding this transient term into Equation (6), it can be written in the temporal domain as

(10) $$I^{2}R_{T0}(1+\alpha_{T}(T_{H}-T_{\infty }))=\rho VC_{P}\frac{dT}{dt}+k_{f}A_{s}\left(\frac{T_{H}-T_{\infty }}{L}\right)$$

The term $\rho VC_{P}$ can be considered as the thermal mass (analogous to electrical capacitance in a lumped system model) and consists of the thermal mass of the solid hot wire and the fluid. However, the thermal mass of the hot wire is much larger than that of the fluid and so the latter can be neglected.

In Equation (10), the left-hand side represents the rate of heat generation due to electrical phenomena whilst the right-hand side describes the transient temperature change in the wire $\rho VC_{P}\frac{dT}{dt}$ coupled with the conductive heat transfer into the $\left(k_{f}A_{s}\left(\frac{T_{H}-T_{\infty }}{L}\right)\right)$. This shows that $k_{f}$ is the property of the gas medium that effects the heat dissipation from the hot wire. The time-resolved temperature solution can be obtained from Equation (10) by using the solution of $\frac{dT}{dt}+\frac{T}{\tau }=0$ being $T=ae^{-t/\tau }$, resulting in

(11) $$\left(T_{H}-T_{\infty }\right)=\frac{I^{2}R_{T0}L}{k_{f}A_{s}-\alpha_{T}I^{2}R_{T0}L}\left(1-e^{\frac{-t}{\tau }}\right)$$

where the heating time constant, τ, is defined as

(12) $$\tau =\frac{C_{p}L\rho V}{k_{f}A_{s}-\alpha_{T}I^{2}R_{T0}L}$$

**Figure 7:**
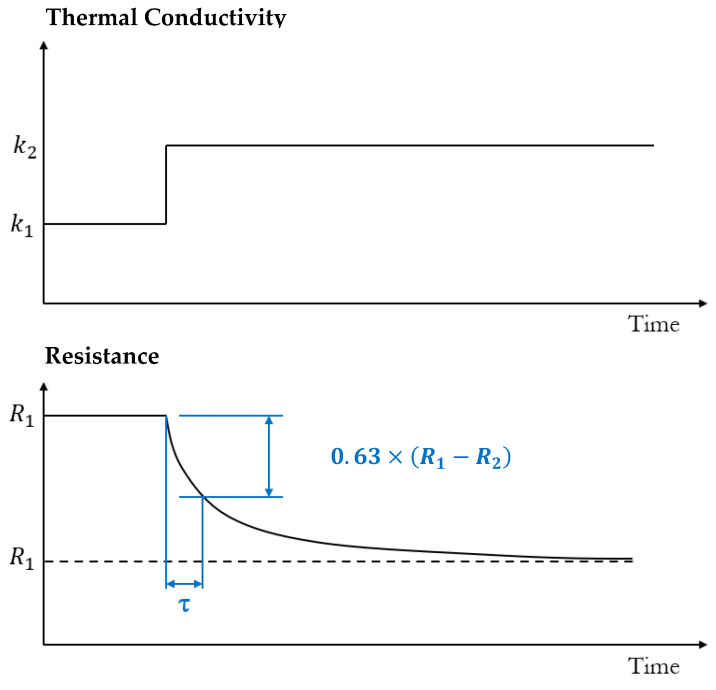
Transient response of the resistance of a constant current hot wire to a change in fluid thermal conductivity.

From this equation, it is shown that the heating time constant is inversely proportional to the thermal conductivity of the gas, illustrating the fundamental methodology of measuring thermal conductivity through this method. The hot wire resistance is dependent on the average temperature and thus its resistance is expected to behave exponentially during heating. The time constant process is graphically illustrated in Figure 7.

It should also be noted that because the thermal conductivity of the gases themselves are strongly temperature dependent, this only holds true for smaller temperature changes.

A second embodiment of the THM includes the hot wire and a temperature sensor that is located a fixed distance away from the hot wire. This temperature sensor then measures the increase in the gas over time when it is heated by the hot wire. In order to disregard the distance and thermal diffusivity, a temperature difference is measured at two points in time. For this to be valid however, the hot wire and distance to temperature sensor must be small as well as not taking the initial temperature measurement when the hot wire is switched on.

Similar to DC operation, a proportion of the heat transfer will be lost through the hot wire into the supporting solid structures. It is once again of paramount importance to minimise this conductive loss in order to access optimum performance from this method.

Due to the inherent nature of the THM, measurement time is very short and power consumption low, highlighting two of the key advantages of this method. This transient nature also leads to the drawbacks that in order to accurately capture the required data, sophisticated electronics and post-processing techniques need to be employed.

#### 2.2.2. 3-Omega Method

A popular technique for the electro-thermal characterisation of materials is the 3*ω* method. This method was originally developed to measure the thermal conductivity of metal filaments that were used in incandescent lightbulbs [13]. Over 70 years later the method was used for measuring the thermal diffusivity of liquids by Birge and Nagel [14] and the thermal diffusivity and thermal conductivity of dielectric solids by Moon et al. [15]. The first use of the 3*ω* technique for measuring thermal conductivity in solids was carried out by Cahill in 1987 [16] and used a microfabricated metal deposited onto the structure under investigation, which was used as the heater. With the onset of MEMS, this technique became very popular for measuring the thermal conductivity of various materials used in micromachining, especially dielectrics [17] (p. 750), [18,19,20,21,22] that have low values of thermal conductivity.

**Figure 8:**
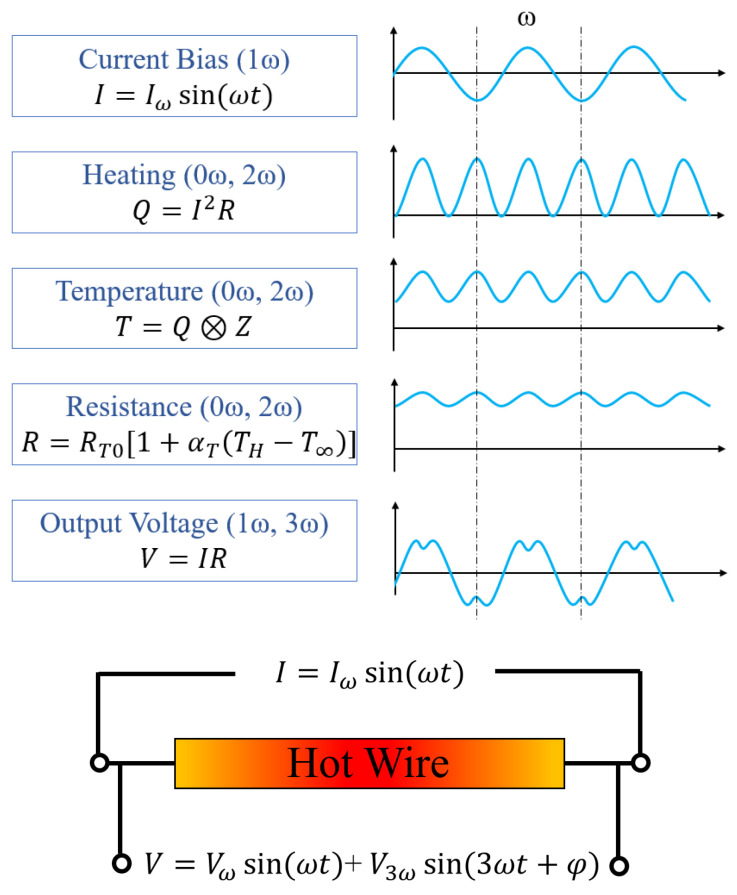
The relationships between sinusoidal excitation, heating and voltage measurement of the hot wire, used to help illustrate and understand the 3*ω* technique.

The 3*ω* technique was developed to measure the thermal conductivities of fluids by Cahill and Mingo [23,24] in which the described method can measure both thermal conductivity and thermal diffusivity. The basic principles of the 3*ω* technique are now described with the illustrative aid of Figure 8. 

The 3*ω* technique is transient by the use of an Alternating Current (AC) excitation signal. This excitation signal is passed through the resistant dependent material (hot wire) which acts as both a heater and a temperature sensor. The hot wire is biased with a current *I*, amplitude of $I_{\omega }$ and frequency of *ω*.

(13) $$I=I_{\omega }\cos\left(\omega t\right)$$

The power dissipated, *P*, by the hot wire is through Joule heating and depends on the current, *I*, and the resistance, *R*.

(14) $$P=I^{2}R$$

This results in the heating of the hotwire displaying a frequency of 2*ω* and gives the instantaneous power when combining Equations (13) and (14) where $R_{T0}$ is the resistance of the hotwire at a reference temperature (typically 25 °C):

(15) $$P={0.5I_{\omega }}^{2}R_{T0}(1+\cos\left(2\omega t\right))$$

Thus, the power can be separated into two components where the first is a constant component independent of time (0*ω*) and the second is an oscillating component (2*ω*).

(16) $$\dot{Q}=P_{DC}={0.5I_{\omega }}^{2}R_{T0}$$

(17) $${\dot{Q}=P}_{AC}\left(t\right)={0.5I_{\omega }}^{2}R_{T0}\cos\left(2\omega t\right)$$

The average power dissipated through the heater is known as the root mean square (rms) power, which is half of the power dissipated by a DC current of the same amplitude. The rms power is defined as:

(18) $$P_{rms}={I^{2}}_{h,rms}R_{T0}$$

The steady-state harmonic temperature oscillations in the hot wire produce harmonic variations in the resistance of the hot wire given by [25]

(19) $$R(t)=R_{T0}(1+\alpha_{T}{∆T}_{DC}+\alpha_{T}\left|{∆T}_{AC}\right|\cos\left(2\omega t+\varphi \right))$$

where ${∆T}_{DC}$ is the steady-state temperature increase due to $P_{rms}$ dissipated, ${∆T}_{AC}$ are the temperature oscillations due to the sinusoidal power component and *φ* is the phase lag between the temperature oscillations and the excitation current.

The output voltage can be described by Equation (20) where the 3*ω* voltage component results from the multiplication of the oscillating current with the periodic heater resistance

(20) $$V(t)=IR_{T0}\left[(1+\alpha_{T}{∆T}_{DC}\cos\left(\omega t\right)+0.5\alpha_{T}\left|{∆T}_{AC}\right|\cos\left(\omega t+\varphi \right)+{0.5\alpha }_{T}\left|{∆T}_{AC}\right|\cos\left(3\omega t+\varphi \right)\right]$$

From this, the in-phase and out-of-phase temperature oscillations can be measured by means of the voltage signal [26] and provide information about the thermal environment.

Both the magnitude and phase of the temperature oscillations vary with excitation frequency due to the thermal properties of the specimen and the thermal conductivity can be calculated by measuring the slope of the in-phase temperature difference versus the logarithm of the excitation frequency.

If a distance, $L_{D}$ is introduced into the system, the diffusion of the temperature oscillations through the specimen will affect both their magnitude and phase. A thermal diffusion time, $\tau_{D}$, is the required time for the thermal wave to propagate $L_{D}$ and is given by the equation:

(21) $$\tau_{D}=\frac{{L_{D}}^{2}}{\alpha }$$

Thus, depending on the decay of the diffusing oscillations, the thermal diffusivity can also be calculated.

In fact, in order to help separate thermal conductivity and thermal diffusivity in the measurement of gases, excitation frequency can be used advantageously. The penetration depth of the thermal oscillations, $L_{P}$, is given by

(22) $$L_{P}=\sqrt{\frac{\alpha }{\omega }}=\sqrt{\frac{\alpha }{2\pi f}}$$

telling us that at low modulation frequencies the penetration depth is large. This means the thermal wave penetrates far into the gas and the system experiences quasi-steady heating where the thermal transport is dominated by the thermal conductivity of the gas. This means that the amplitude of the 3*ω* signal can be used to distinguish gas mixtures by their thermal conductivity alone. At higher frequencies the penetration depth is much smaller and the phase lag is dictated by the effective thermal diffusivity of the gas and a characteristic frequency can be calculated to find maximum phase lag sensitivity [27].

Due to the nature of the 3*ω* technique, a lock-in amplifier is necessary in order to extract the amplitude and phase shift of the sinusoidal signal, highlighting the main drawback of the requirement of complicated post-processing techniques and electronics. However, this modulation provides the opportunity to increase selectivity and sensitivity in gas measurement systems and lock-in amplifier integrated chips are now available at low cost when using a standard CMOS process.

#### 2.2.3. Thermal Resonant

Electrothermally actuated MEMS resonators have been utilised as a base for many biological, chemical and gas sensors. The operation principle of such devices is based on tracking the shift in frequency of the fundamental mode due to external stimuli. In the case of gas sensors, the overwhelming majority of reports use a thin layer of selective material. Such materials have an affinity for particular gases, such as gold for mercury detection [28], carbon nanotubes [29] for carbon dioxide and Metal Organic Frameworks (MOFs) for VOCs [30]. A detailed review on resonant sensors for environmental, chemical and biological sensing can be found here [31].

Pure thermal conductivity-based resonance technologies are very minimally researched in comparison with their absorption-based counter parts, which is largely because of the lack of sensitivity displayed in these devices when there is no mass alteration from analyte-binding. However, if this issue can be resolved then sensors without absorption-based layers exhibit superior lifetime and stability.

Hajjaj et al. [32] operate a doped-polysilicon resonator near to its buckling point in order to maximise the first resonant mode variation to cooling and heating effects. When altering the gas thermal conductivity (with respect to nitrogen), the values of resonant frequency change and by tracking this frequency shift, gas concentration can be determined. Experimental and simulation results showed that the resonant frequency was a function of gas concentration for hydrogen, methane, propane and carbon dioxide without the need of specific functionalisation for each gas. The report described an improvement on previous attempts but does not investigate the Limit of Detection (LoD) for the gases.

## 3. Signal Processing

All TCD’s have an actuator (a device for heating) and a temperature sensor (for detecting the physical parameter). In many cases, it is often convenient to combine them into a structure that can do both. However, they can be split up, which leads to many types of temperature sensing technologies and methods. When separate structures, both can be optimised for their specific function.

### 3.1. Thermal Transduction Methods

In order to realise thermal conductivity based gas sensors, small changes in thermal conductivity must be measured. Considering the thermal conductivity values of air and CO_2_ at 300 K are similar (26.2 mW/m∙K and 16.8 mW/m∙K, respectively), a 1% change in CO_2_ concentration would result in a sub −0.4% change in thermal conductivity. This precision requires a high-resolution temperature sensor to capture the small changes in heat transfer equating to these levels.

Micromachined thermal gas sensors can employ a variety of physical transduction principles that convert from the thermal domain into the electrical domain, in order to be connected to a circuit and measured. This section provides an overview on the different methods of measuring temperature that are commonly employed in gas sensing applications and techniques. A comparison of the three main methods for temperature measurement is presented in Table 2.

#### 3.1.1. Thermo-Resistive

The most common method of temperature measurement comes from a thermo-resistor (sometimes known as thermo-anemometer). Here, a change in temperature results in a resistance change, and the corresponding change in power can be calibrated to the temperature of the resistor. These are the most popular type of thermal sensor due to their ease of fabrication and simple operation.

The most common thermo-resistive devices are Resistive Temperature Detectors (RTDs) and thermistors. The difference between the two is from the material they are made of. Thermistors are made with polymer or ceramic materials whilst RTDs are made from metals or semiconductor material. The by-product of this difference means that thermistor commonly have a negative TCR, i.e., their resistance goes down with temperature whilst RTDs have a positive TCR. It is for this reason that in MEMS devices almost all thermo-resistive temperature detectors are RTDs, and most commonly CMOS metals.

#### 3.1.2. Thermo-Electronic

Thermo-electronic transduction uses transistors or diodes as the thermal sensing element. Diodes can be used for temperature sensing due to the strong temperature dependence of their forward bias voltage drop. Many different semiconductor materials have been reported in literature for diode temperature sensors (silicon, germanium and selenium are some examples). The earliest use of silicon diodes as temperature sensors was reported by Harris [33] and McNamara [34]. More recently, designs have used the thin silicon layer in commercially available SOI processes to fabricate the diodes. A review on diode-based temperature sensors can be found here [35].

Some research has also been carried out into using transistors as temperature sensors. Especially when good long-term stability and high sensitivity over a limited range (−50 °C to 125 °C) is wanted. These favourable qualities are due to a highly predictable and time-independent base-emitter voltage relationship with temperature [36].

#### 3.1.3. Thermo-Electric

**Figure 9:**
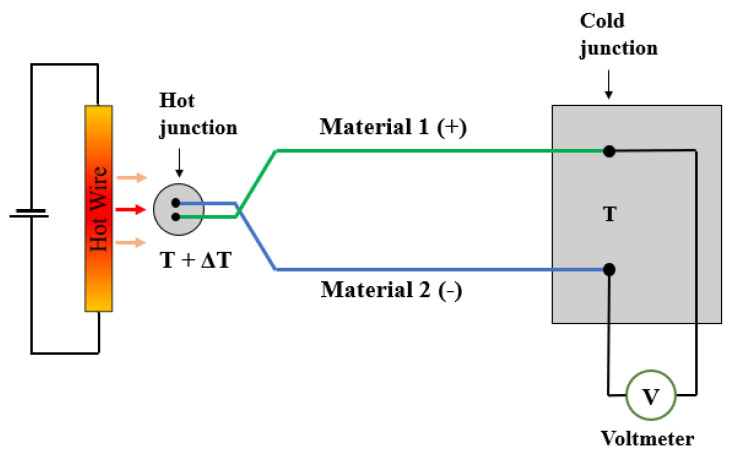
Illustration of the working principal of a classical thermocouple.

Thermo-electronic transduction detects the thermal changes using thermopiles. Thermopiles are multiple thermocouples connected in series, and thermocouples are based on the thermoelectrical effect generated in the junction of two dissimilar metals (or metal/silicon junctions). These must be operating in conjunction with the heating element, thus requiring an extra component for the sensing system [37,38]. Figure 9 illustrates the working principal of a thermo-couple whereby two dissimilar metals (or semiconductors) are electrically connected at one end. Applying heat to the junction of the two materials produces a voltage between the two wires, and this voltage is proportional to temperature. The fabrication of thermopiles is more complicated because less conventional materials are used for their fabrication; however, CMOS compatible fabrication is still possible.

The thermoelectric effect (Seebeck effect) enables a thermocouple to provide higher sensitivity and voltage output with less offset and drift. The voltage output of a thermocouple, $V_{tc}$, is given by the following equation:

(23) $$V_{tc}=\alpha_{tc}∆T_{tc}={(\alpha }_{tc,a}-\alpha_{tc,b})(∆T_{tc})$$

where $\alpha_{tc}$ is the thermocouple Seebeck coefficient, $\alpha_{tc,a}\;\&\;\alpha_{tc,b}$ are the Seebeck coefficients of the two dissimilar materials and $∆T_{tc}$ is the temperature difference between the hot and cold junctions of the device.

Thermopiles are constructed from thermocouples in series, which results in a voltage output is summed and increased over that of a single thermocouple. However, thermal conduction between hot and cold junctions and Johnson (thermal) noise increases with the addition of multiple thermocouples, thereby inhibiting sensor performance. It is for this reason that high thermal isolation is desired in order to maximise the temperature difference between the hot and cold junctions. In fact, semiconductor thermopiles are more sensitive than their metal counterparts because of their high Seebeck coefficients, which can also be tuned with dopant type and concentration. For micromachined gas sensing solutions, polysilicon and metals are commonly used for thermopiles.

## 4. Parasitics and Interference

Parasitics are undesirable elements that are unwanted and may affect the expected performance or response. This interference is a problem for many sensors and if the effect of the parasitic is large enough, there needs to be consideration and most likely compensation to retain the accuracy of the device.

Thermal gas sensors are based on heat transfer, and thus any physical phenomena that may affect the transfer of heat can interfere with the signal. The following section outlines some of the most common parasitic effects.

### 4.1. Temperature

In almost all applications in which gas sensors are used, there will be fluctuations in ambient temperature. This could vary from gas detection for Indoor Air Quality (IAQ) where the environment will be controlled and only vary between 15 °C and 25 °C to extreme temperature applications such as gas turbine power plants, nuclear power plants, internal combustion engines, food storage or cryogenic chambers.

For both steady-state and transient methods of running a thermal gas sensor, the response depends on the ambient temperature, which is defined as $T_{\infty }$ in Equation (2), Equation (11) for the transient hot wire method and as $R_{T0}$ (resistance at ambient temperature) in Equation (19) for the 3-omega method.

These equations highlight the importance of temperature compensation in thermal gas sensor systems. For example, the difference in thermal conductivity in air from 0 °C to 60 °C at atmospheric pressure is 4.4 mW/m·K, as taken from the data underlying Figure 3. The difference in thermal conductivity of 100% air and 99.9% air and 0.1% H_2_ (i.e., 1000 ppm hydrogen is 0.04 mW/m·K). This means that when detecting 1000 ppm of hydrogen, if the ambient temperature changes by 60 °C then the parasitic response will dominate the gas response by two orders of magnitude, thus highlighting one of the axioms of thermal gas sensor deployment; temperature compensation.

The most common method of compensating for temperature is to use a reference technique whereby there is one element exposed to the measurand environment and a second element that is exposed to a controlled environment. This means that when ambient temperature changes, both elements change temperature but when the environmental gas composition is changed, only one of the elements will change its reading. Resulting in a delta response that is compensated for temperature. This is very common is pellistor-like systems and GC applications where the two elements are often connected in a Wheatstone bridge, as shown in Figure 5 [39,40,41,42,43].

Some variations on reference cell temperature compensation include Xue et al. [44] who design an analogue circuit with a compensation resistor to make the voltage temperature coefficient between sensitive element and reference element same so as to realise temperature drift compensation. Gardner et al. [45] present a sensor where there are two elements on the same membrane which are thermally matched, a difference in CO_2_ response is then generated by using holes through the membrane to force each element to interact with the atmosphere differently.

A method which is commonly used in commercial devices is to have a separate temperature sensor and algorithmically compensate for temperature using post-processing methods, either in the hardware or software.

### 4.2. Humidity

Similarl to temperature, many applications will have varying humidity in the environment that is to be measured. Humid air will have different thermal transport properties to dry air and will affect the thermal conductivity, changing the response of the device. An investigation into the thermophysical and transport properties of humid air at temperature range between 0 °C and 100 °C can be found here [46].

**Figure 10:**
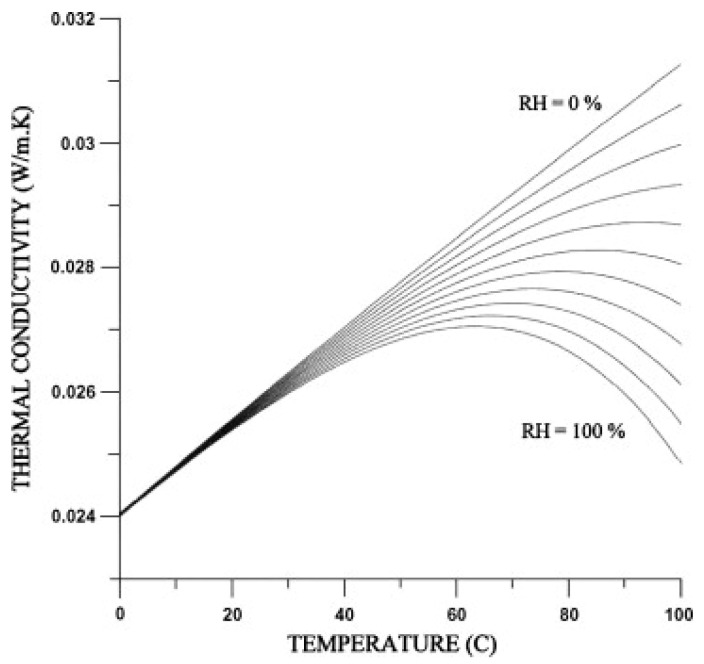
The thermal conductivity of moist air as a function of temperature with the relative humidity as a parameter ranging between dry air (top curve RH = 0%) and saturation conditions (lower curve RH = 100%) in 10% steps. Figure from [46].

Figure 10 shows how thermal conductivity of air changes with temperature, for different values of relative humidity. It shows that at higher temperatures, there is a much stronger effect of humidity, which is due to air being able to hold much larger amounts of water vapour at higher temperatures. This means that at higher temperatures, the absolutely humidity is much higher for the same relative humidity. Humidity compensation becomes especially important as temperature increases for this reason.

Humidity compensation is minimally researched with few investigations in the literature. The most common method of compensating for it is to integrate a separate humidity sensor into the system and algorithmically cancel out the effect through firmware or software [47,48].

Another consideration with humid air is damage to the sensor. If there is a drop in temperature in a system, humid air can condense and leave water droplets on the system. Designers need to be considerate that:

- Condensed water droplets will not damage the sensor itself, or cause accelerated aging of the device.
- If there is potential for condensation in a system, there must be a method of purging or removing this condensation.

### 4.3. Pressure

**Figure 11:**
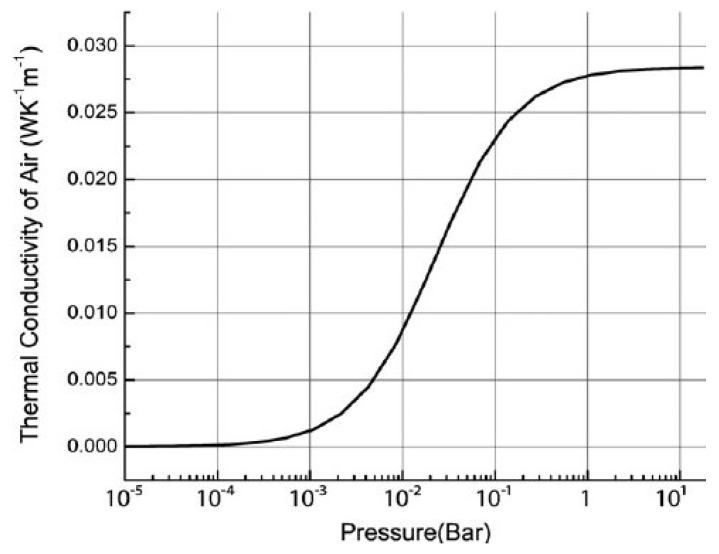
Thermal conductivity of air as a function of pressure. Figure from [49].

Another parasitic that can change the response of thermal gas sensors is ambient pressure. Figure 11 shows the change in thermal conductivity of air as a function of pressure, highlighting the need to consider compensation, especially in the regions between 10^−3^ Bar and 10^−1^ Bar. The effect of pressure is much smaller when pressure is >1 Bar, demonstrating that careful consideration of system deployment conditions needs to be taken.

In addition to the change in thermal conductivity of the gas due to pressure, atmospheric pressure changes can affect the device itself. For example, if there is a device that has a sealed portion within the MEMS design, change in atmospheric pressure can cause pressure differences and apply mechanical stress to MEMS structures. This can cause reading changed through elastic deformation, reading drift through plastic deformation and catastrophic failure. A solution for membrane devices is to create holes in the membrane for pressure equalisation [5].

The most common method for compensating ambient pressure is to integrate a reference pressure sensor in the system and algorithmically compensate in the system firmware or software. Pressure sensors are widely commercially available at low costs for system integration.

### 4.4. Flow

Thermal gas sensors are based upon the same principal as thermal flow sensors. If there is flow in the system, or more specifically across the active element, forced convection will change the heat transfer and alter the response seen by sensor. The temperature of a heated element is calculated through its resistance; however the dominant form of heat transfer of this element becomes forced convection, which is much stronger than conduction/natural convection.

If we look at Equation (3), $h_{T}$, is the heat transfer which in this case is dominated by forced convection. The dimensionless heat transfer coefficients Nusselt number and Reynolds number can be used to substitute out the heat transfer coefficient in order to see the relationship of current to velocity, which is referred to as King’s Law:

(24) $$I^{2}=\frac{R-1}{R}(A_{1}+A_{2}U^{0.5})$$

where *R* is the ratio of resistances $R_{S}/R_{T0}$, $A_{1}$ and $A_{2}$ are constants to be determined and U is the fluid velocity.

This analysis has some simplifications that include neglecting axial heat conduction in the wire, heat loss through the structural support system, dynamic response of the heated wire and effects of the measurement circuitry. It is also shown that the quadratic relationship breaks down with highly turbulent flows and MEMS devices. A review on micromachined thermal flow sensors can be found here [50].

It is often necessary to have a constant system flow rate or to measure the flow rate and calibrate each sensor for the flow rate range, which add system complexity and cost. The most common approach is to remove any flow around the sensor on the package level. This is achieved by ensuring there is not a flow path around or across the sensing element. This can be seen in various commercial devices that have a hole or series of holes in the package, forcing the atmosphere to change in the package environment through diffusion. If the system is not designed to interact with the target gas through diffusion careful consideration needs to be made on MEMS and package design.

There have been some other investigations into reducing the effect of flow on the gas response. Graaf et al. have shown a micro thermal conductivity sensor where the effect of flow is reduced by between 4–15 times [51]. This is achieved by have a reference device with a larger cavity. The active device with a smaller cavity will be more strongly affected by the thin layer of gas in this device compared with the larger cavity providing partial compensation for flow. Hepp et al. have presented a thermal conductivity sensor that uses a constant power mode and runs in a region of flow independence [52]. Romero et al. present a calorimetric flow sensor that can also determine the thermal conductivity of gases under flow conditions [53]. The sensor uses DC excitation to measure the flow rate and AC excitation to infer the thermal conductivity at the flow rate. Gardner et al. demonstrated a device with three active elements with different responses to flow and thermal conductivity. Artificial neural networks were then used to map the multi-parameter response to output both flow and thermal conductivity. Wang et al. have presented a thermal conductivity sensor where they have ensured the heated element is exposed to gas by diffusion, this is by putting it out of the main flow path [54].

### 4.5. Gas Mixtures

The thermal transport properties of gases change when there are mixtures of gases present in the same atmosphere. There is a large diversity of formulas and empirical analysis on mixing rules for these transport properties which has resulted from there not being a unified theory of thermal conductivity in gas mixtures.

There were many works in the 1950’s and 1960’s using theory to create approximate formulas to describe the change in thermal transport of gas mixtures [55,56,57,58,59]. Mason and Saxena [58] describe a difference between monatomic and polyatomic gases which is due to the mechanism of energy transfer being different. Therefore, hydrogen in gas mixtures will behave different due to its small size allowing a stronger interaction in collisions. This results in a slightly higher thermal conductivity of hydrogen and its mixtures than those derived from theory based on all gases. They also propose a semi-empirical model that takes into account the effect of viscosity. It is stated by Zhulov et al. [60] that the semi-empirical mixing rules proposed by Mason and Saxena were considered the best for predicting thermal conductivity of gas mixtures for rocket combustion chambers. Recently, Azmi et al. [61] give a review on the thermal conductivity and dynamic viscosity of nanofluids, whereby a nanofluid contains some dispersed particles of less than 100 nm.

It is extremely important to know that the thermal transport properties of gases do not scale linearly. This must be included for numerical analysis as well as throughout algorithms of multi-gas detection systems, especially in an age where the limit of detection is being constantly reduced.

## 5. Applications

This section contains a brief description of the main commercial applications in which thermal conductivity gas sensors can be found. There has been a resurgence of interest in MEMS thermal conductivity gas sensors in industry, with the development of commercial products being seen in the last 5 years from worldwide leading sensor companies such as Bosch, Sensirion, TDK, SGX Sensortech, and AlphaSense Ltd.

### 5.1. Gas Chromatography

The Thermal Conductivity Detector (TCD) is often referred to as a katharometer and is used as a chemical specific detector most commonly in Gas Chromatography (GC). Devices designed to measure thermal conductivity have been described since the 1880s, and in the early 1900s they were used in a variety of applications (mostly gas analysis in chemical industries). This established technique was then integrated into GC systems in early developments of the technique, especially with GC systems using helium or hydrogen as the carrier gas. By the end of the 1950s, the TCD was a mature detection system for gas-solid chromatography.

Despite many attempts to improve it, the sensitivity of the TCD continues to be its most limiting factor. In fact, TCD systems were commonly replaced with the introduction of ionisation detection systems, which offered improved sensitivity. The TCD, however, remains very useful in the determination of gaseous substances that are difficult to detect by other means, such as COS, H_2_S, SO_2_, CO, CO_2_, NO, and NO_2_. Table 3 provides some typical characteristics for methods of detection that are used with GC systems. The first noticeable benefit of the TCD methods is that it is a universal detector, as opposed to a selective detector. This means it will respond to any solute presented to it in the system and highlights its advantage over ionising techniques that are mostly selective. FID is commonly used in petroleum analysis as it responds selectively to hydrocarbons. Among the selective detectors we also have element sensitive for AED, which means it will respond only to a particular element of interest.

The Limit of Detection (LoD) is sometimes referred to as the Minimum Detectable Level (MDL). This is the minimum mass (or concentration) of a substance that can be measured by the detector. This can be determined from the measured sensitivity and noise, thus highlighting that with sensitivity enhancements comes the added benefit of lowering the LoD.

With the onset of micromachining and MEMS fabrication technology, the miniaturisation of TCDs (into micro-TCDS [µTCDs]) has received considerable attention in order to create miniaturised or micro-GC systems (µGC). µGC systems will provide the advantages of lower cost and power consumption whilst also increasing sensitivity, accuracy and response time. In addition to this, the smaller size will enable portable capability, allowing entrance into other fields such as health services, industrial process control and geological exploration. It should also be noted that TCDs are more suited for miniaturisation due to their sensitivity being related to concentration mixture and not total mass of the sample (like FID).

µTCD for µGC literature is limited in comparison to other gas sensor technologies, with most literature directed towards improving the sensitivity of the device. Conventional bench-top gas chromatography systems have need of being replaced by μGC due to their ability to offer a fast, on-site, low-power, portable system that can provide real-time monitoring for quantification and identification analysis of complicated gas mixtures. Therefore, many efforts have been performed to construct μGC systems. Among the first efforts to miniaturise this technology came from the University of Stanford in 1970 [62] where the first silicon based thermal conductivity sensor was fabricated separately and integrated onto the substrate wafer. This TCD device had problems with sensitivity and many more recent efforts have been carried out to try and improve this.

Most reported µTCDs are suspended bridge structures, to maximise interactions with the flow of gas. The mechanism of heating is most commonly thin-film deposited metal [40] or polysilicon [63,64]. There has been some investigation into optimising structure, including shaped heater [65] and non-suspended structures [41,66] as well as CMOS compatible designs [67].

Cruz et al. microfabricated a TCD to be used in conjunction with a surface acoustic wave detector in a microChemLabTM system [68]. Here, the geometry of the TCD was optimised and investigated in different orientations to the flow. Sun et al. used highly isolated resistors with a Wheatstone bridge [69]. A polydimethylsiloxane (PDMS) membrane was used instead of the conventional glass to seal the μTCD at the room temperature and the device was shown to detect CH_4_ with a detection response of 500 ppm and response time of 30 s. Sun et al. also used a µTCD device within a µGC system in order to detect the VOCs of benzene, toluene, and styrene in less than 3 min. Narayanan et al. have presented a monolithic integration of a µTCD embedded within the separation column and have outlined their systems advantages and disadvantages when compared with three other µGC systems that use chemical based detection mechanisms [66,70]. A collection of recent updates on µGC systems is given by Qu and Duan [71].

### 5.2. Flammable Gas Sensing

Thermal conductivity sensors are especially suited to detect hydrogen, as hydrogen possesses the highest thermal conductivity of all known gases. It is therefore possible to detect low concentrations of hydrogen in air by the increase in thermal conductivity of a hydrogen-air mixture. Thermal conductivity sensors thus seem to be the first choice for safety applications such as hydrogen detection in automotive combustion or fuel cell application. In such systems leakage of hydrogen needs to be detected before hydrogen builds up an explosive mixture with the ambient air, the lower explosive limit being 4 vol.% H_2_ in air. Fast response times and low power consumption are desired for such systems. Therefore, micromachined sensor elements are very attractive for this application and are often used in conjunction with catalytic detectors when they are used above the LEL. TCDs are mostly used in the detection of hydrogen and methane in this sector, due to their thermal conductivity values being very different to air. An evaluation of commercial hydrogen sensors and their selectivity can be found here [72], which compares TCD with the other commonly available methods. A solely TCD device for the detection of hydrogen and methane is commercially available from the company SGX SensorTech.

Applications where TCDs are used to measure hydrogen include hydrogen-cooled generators used in power-plants, hydrogen as a clean and renewable alternative to carbon-based fuels where safe and efficient deployment is crucial [73], automotive combustion/fuel cells [47,74] and the hydrogen leak detection [75,76,77], this has yielded investigation into improving TCDs specifically for hydrogen detection [78]. The clean burning process of methane makes it an attractive fuel, however, due to its gaseous form at room temperature it is transported by pipeline or (Liquified Natural Gas) LNG carrier and it is thus necessary to monitor the safe use and transport of this gas resulting in the development of TCD devices for the detection of methane [79]. Some research has gone further to investigate the ‘Methane Number’ of natural gas, which helps engine operation and reduce degradation [80].

### 5.3. Medical

The biomedical sensor is rapidly growing with the development of smart, micromachined sensor systems. Current diagnostic methods, such as blood tests, are slow and invasive and thus highlight the need for micromachined, quick and non-invasive gas sensors.

One such solution is designing sensors for human breath analysis. This ability of gas sensors to detect diseases is, in part, due to the nature of human breath, which along with containing the expected gases such as nitrogen, oxygen, water vapour, and carbon dioxide also consists of other trace species including ammonia (833 ppb), acetone (477 ppb), ethanol (112 ppb), acetaldehyde (22 ppb), and propanol (18 ppb) with some even at concentrations as low as a few parts per trillion [81,82]. When the concentrations of these VOCs significantly change, it can be an indicator of a medical problem. Some examples include acetone as a marker for diabetic persons [83], CO for detecting smokers and nitrous oxide for asthma. The concentration of these biomarkers is extremely low and thus renders using thermal conductivity gas sensors very difficult. If the sensitivity and selectivity of thermal conductivity devices can be improved to work in these ranges, the technology will be able to compete commercially. Until then, TCDs will be used in applications where higher detection limits are required.

Some examples of thermal conductivity being used in medical applications are the measurement of xenon for use as an anesthetic [84,85].

### 5.4. Other

Quantitation of ethanol and water is important in manufacturing, processing, and quality control of many commercial products. Consumer products, such as beer, wine, liquor, mouthwash, and flavour extracts, contain ethanol and water usually in concentration ranges of 4–50% (*v/v*) ethanol and 50–96% (*v/v*) water. There is a need for a simple, rapid, cost-effective, precise, and accurate method for the quantitation of both ethanol and water that will also enhance throughput and efficiency. Some work has been carried out on using TCD detectors for the quantification of water [86,87,88]. Weatherly et al. used a TCD device to simultaneously quantify ethanol and water concentration in commercial products [89].

**Figure 12:**
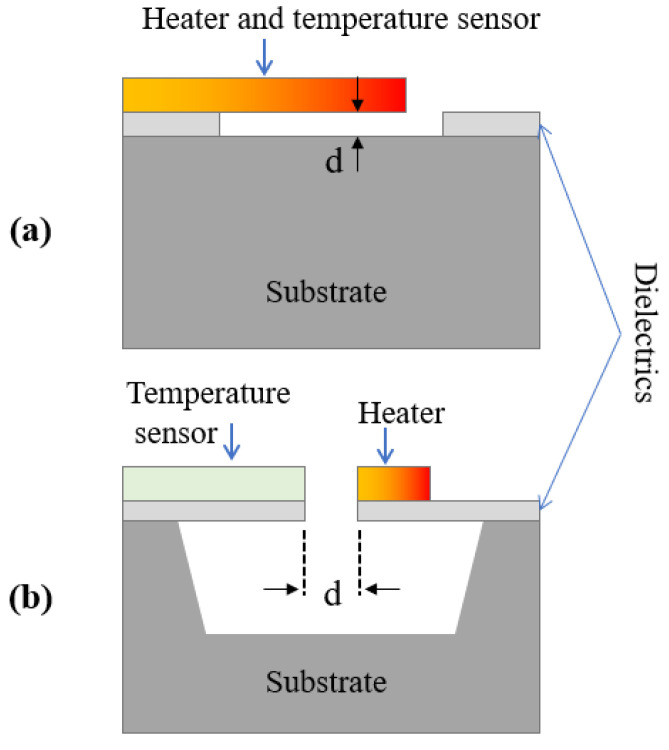
The structure of a micro-Pirani sensor with both heating methods that include (**a**) self-heating and (**b**) mutual heating.

Miniaturised vacuum sensing [90] is essential for the correct operation of many manufacturing apparatuses, especially in the field of integrated circuit fabrication. Vacuum monitoring is also of great importance when a condition of medium or high vacuum is required to guarantee effective thermal insulation, such as in cryogenic systems and thermodynamic solar power plants [91]. Since the first pioneering works in as early as 1906, many MEMS Pirani sensors have been proposed [92,93] and demonstrated, resulting in a viable approach to reduce cost and integrate the sensor with read-out electronics [94,95,96]. This miniaturisation has allowed an increase in device sensitivity due to the small air gap that can be accurate fabricated with micromachining technology. Figure 12 shows an example of a micro-Pirani sensor with the two most commonly used heating configurations [97].

The following describes the basic phenomenon around the Pirani type pressure sensors. If the two parallel surfaces are considered at temperatures *T*_1_ and *T_2_* and are separated by the distance *d*, which is filled by the gas under investigation. The heat transfer $\dot{Q}$ from the heated surface to the substrate can be approximated by the linear relationship:

(25) $$\dot{Q}=G_{gap}(T_{1}-T_{2})$$

where $G_{gap}$ is the thermal conductance of the gap across the distance *d*, in the investigated gas. The gap thermal conductance has a dependence on the gas pressure, *p*, given by

(26) $$G_{gap}=\frac{G_{0}}{1+\frac{p_{tr}}{p}}$$

where $p_{tr}$ is the transition pressure, which depends on gas type and surface composition.

The most widely used application of the Pirani gauge is for the measurement of pressure in vacuum packaged MEMS devices such as resonators, gyroscopes and devices that require thermal isolation [98,99]. Two common alternatives for measuring in situ pressure are the helium leak test and Q-factor variations using inertial or resonant devices. Helium leak tests require expensive equipment and have long measurement times whilst Q-factor based devices have longer measurement times alongside more complex calibration and data processing necessities.

## 6. Recent Developments

The following section provides an overview of recent literature surrounding thermal conductivity gas sensing.

### 6.1. Steady-State Operation

It is known that the defining limitations of thermal conductivity sensors with steady-state operation comes from their poor sensitivity and selectivity. This is especially true for the detection of two gases that have similar thermal conductivity values, such as air and CO_2_. With the advancing of this technology and the improvement of sensitivity, applications in the measurement of such gases can be opened to this technology that is inherently cheaper and less complex, with greater response times. For these reasons, most literature looks at improving gas sensitivity by various means. This section will highlight the state-of-the-art research performed on micromachined thermal conductivity sensors that are operated under steady-state conditions.

The first point of optimisation comes from the heated element itself, which is directly accountable for the heat transfer magnitude between the sensor and the measurand. Firstly, various materials have been used such as platinum [100], tungsten [5,101,102], polysilicon [64,103], carbon nanotubes [104] and other carbon materials [105], including a gold-coated carbon nanoribbon-based heater [106]. It should be noted that carbon nanotubes must be locally grown onto an already fabricated device, rendering this design not CMOS compatible and hence not yet a commercially viable solution. Table 4 shows the four most common metals used for the metal layer within the CMOS MEMS process and their various material properties. Therefore, the majority of devices, especially with high volume capability, will be made from these metals.

Special attention should be paid to the Temperature Coefficient of Resistance (TCR) because this value defines the amount that the metal’s resistance changes with temperature and thus defines its sensitivity. It can be seen that tungsten has the highest TCR and will display the largest sensitivity with the same change in temperature; after tungsten are aluminium, gold and platinum, respectively.

Platinum shows the lowest thermal conductivity, which is beneficial because this material will have the least conduction loss through the tracks and into the substrate of the device. By a considerable margin, the highest melting point is shown in tungsten, which means higher reliability and also provides the possibility of running the heater at higher temperature, thus increasing the potential sensitivity (see Equation (2)). Electrically, platinum has the lowest conductivity, which will result in better power consumption due to the higher electrical resistance yielding more efficient heating up of the resistor. This also offers better potential for further miniaturisation of the sensors. Electrically, platinum is followed by tungsten, and then by aluminium and gold. It is from this table that the front-running CMOS compatible metal is tungsten. Tungsten is used as a high temperature metal or as a popular way to make vias between different metal levels in the CMOS process. Platinum is also a good choice, but platinum is a dopant in semiconductors, and as gold can only be used post-CMOS and not part of the CMOS sequence.

Another important factor is the melting point, of which tungsten has the highest. This allows this material to be operated at much higher temperatures. Higher temperatures can help by increasing sensitivity, which is achieved by a larger difference in temperature between the environment and the sensor. Higher temperatures also allow for more choice when applying thermal modulation, helped by gases different thermal conductivity properties with temperature, as illustrated on Figure 3.

Once the resistor material is chosen, the shape of the heated element can be changed to alter the amount of heat transfer acting between the heater and the environment. As signal is proportional to surface area exposed (see Equation (3)). A simple geometry change is shown by [64] where a central portion of the heater is made thinner in order to have a higher temperature more centrally on the heater and thus lose less heat to the surrounding substrate. Simon and Arndt [47] varied the membrane size and showed that increasing membrane size results in the larger sensor efficiency whilst coming at the cost of power consumption and response time.

Some more complicated examples of changing the heater shape include Narayanan et al. [65], who created a coil shaped Cr/Ni based resistor within a µGC system. The coil shape is produced by annealing the thin metal film after it is released. Another example is shown by Feng et al. [100], noticed that suspended wires are not very reliable due to low structural stability and therefore used DRIE to create a suspended cross-mesh structure. This cross-mesh structure acts as a supporting structure to the platinum resistor and increases the reliability by adding structural integrity whilst minimising conductive heat loss. The platinum resistor is a zig-zag shape deposited across the centre of the mesh and the device is shown to detect 20 ppm of the hydrocarbons C14-C16. Another attempt to increase reliability and robustness used a silicon oxide membrane with isolating holes around the heater. This provides thermal isolation of the heater to increase interaction with the atmosphere whilst providing the structural reliability of a membrane [103]. 

Wang and Tang [101] used surface micromachining to create four tungsten micro hot plates in series each of which is suspended by four beams and has a surface area of 60 µm × 60 µm. The micro hot plate is suspended over a surface containing a temperature sensor for measuring the heat transfer through the gas environment. The sensor reported responses to different gas types including ethanol, acetic acid, acetone, ammonia, formaldehyde, and water.

Thermal conductivity sensors are affected by ambient conditions with a particular problem being seen with the variation of ambient temperature. This is because, as shown in Equation (3), the heater response is proportional to the difference between heater temperature and environment temperature. Thus, if ambient temperature changes the heat transfer within the system changes and causes a drift that limits device sensitivity. A common technique to try and nullify ambient temperature fluctuations is using the Constant Temperature Difference method, where the heater is kept at a constant temperature above the environment, which helps to keep the temperature difference the same. This method requires extra temperature sensors in the system that can measure ambient temperature. This technique has been used extensively for thermal flow sensors [107,108,109,110]. Fewer examples of this can be found for thermal conductivity gas sensors. Xue et al. [44] have used the voltage difference between an active sensor and a reference sensor through an analogue circuit to compensate between 0 °C and 20 °C.

Cai et al. [111] report a ratiometric readout circuit in order to measure CO_2_ concentration through thermal conductivity. They measure the ratio between a CO_2_ sensitive element and a CO_2_ insensitive reference element. Their thermal resistance ratio can then be derived and is expressed as the multiplication of the two ratios: the ratio of the temperature difference of the sensitive resistor (∆*T_s_*), temperature difference of the reference resistor (∆*T_r_*) and the ratio of their power differences ((∆*P_r_*)*/*(∆*P_s_*)), resulting in Equation (27):

(27) $$\frac{\theta_{s}}{\theta_{r}}=\left(\frac{∆T_{s}}{∆T_{r}}\right)\left(\frac{∆P_{r}}{∆P_{s}}\right)G_{gap}=\frac{G_{0}}{1+\frac{p_{tr}}{p}}$$

**Figure 13:**
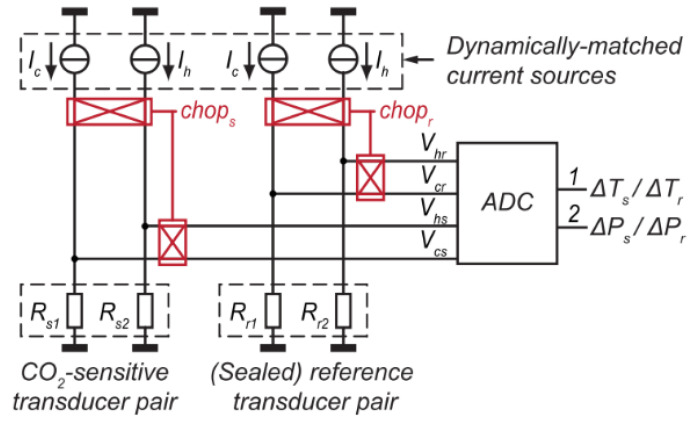
Block diagram of the ratiometric thermal conductivity sensor readout with transducer pairs for baseline-resistance cancellation used in [111].

Using this method helps to relax the need for high-resolution temperature sensors and also reduces the effect of ambient temperature spikes. The inclusion of a power ratio also means the response is less affected by the power noise. A block diagram of their ratiometric readout circuit that uses two transducer pairs is shown in Figure 13.

Ali et al. [112] reported a micro thermal conductivity detector with a platinum heater experimentally tested to detect the three pollutants toluene, ethylbenzene and xylene. The two operating modes of constant current and constant temperature were said to strongly effect the device sensitivity; however, the advantages of both operating modes are not explored beyond this.

Udina et al. [113] used a thermopile as the temperature detector in order to analyse the composition of natural gas using numerical modelling. Thermopiles, due to their ability to detect very small temperature changes, shows “high sensitivity to composition changes.” Experimental results also showed that thermal excitation at different heater voltages can provide additional information due to changes in relative sensitivity to each gas. Further processing and calibration would be required for natural gas component quantification there are some limitations present in numerical modelling that mean experimental evidence would be needed to substantiate this technology in order for it to be considered as commercially viable.

Hepp et al. [52] defined a region of flow independence with gas sensitivity in order to measure gas concentration using a time-independent power excitation. In this setup, the flow rate over the sensor would need to be known in order to measure gas concentration, as the flow independence is only in certain flow rate ranges.

Gardner et al. [114] used a mechanical design to increase gas sensitivity of a tungsten heater as well as post-processing using Artificial Neural Networks (ANN) and liner statistical methods to simultaneously measure percentage CO_2_ (through thermal conductivity) and flow.

### 6.2. Transient Operation

Considerable research has been carried out investigating the transient response of thermal conductivity detectors and trying to utilise the inherent advantages. The following section outlines some of the literature published in the last five years for the two main transient methods of detection.

#### 6.2.1. Transient Hot Wire Method (and Other Temperature Modulation Techniques)

Mahdavifar et al. [64] investigated the transient response of a low thermal mass TCD to an electric square current pulse in multiple mixtures of dilute gases in nitrogen. The thermal element was a 100 µm long by 2 µm wide doped polysilicon microbridge. It was shown that the time constant was a linear function of the thermal conductivity. The level of detection was 25 ppm for helium in hydrogen and 178 ppm for carbon dioxide in hydrogen. Within this report it was also shown that the power consumption for the THM method was much less than when the sensor is driven in dc mode.

Struk et al. [103] fabricated a MEMS thermal conductivity gas sensor based on a heated polysilicon bridge. Three different designs of bridge were investigated that included a rectangle, a rectangle with a thinner section in the middle and a U-shaped structure. It is concluded that longer heaters are more sensitive than shorter heaters because they have a larger surface area to volume ration from which to dissipate heat. The sensors were also operated at different temperatures, which allowed differences in gas thermal conductivity to be identified. The devices limit of detection for carbon dioxide in nitrogen was found to be 3313 ppm.

Randjelovic [115] demonstrated the simultaneous measurement of binary gas mixtures and pressure based on the thermal response time of thermopiles. An analytical model was developed to determine the optimum number of thermopiles and showed that the thermal time constant depended on the geometric design of the sensor. After this, the sensor was shown to measure binary gas mixtures of helium and argon alongside pressure. The binary gas fractions investigated in this investigation are very large, and do not give detail on the accuracy or LoD the device could be capable of.

Cai et al. [116] develop a phase-domain readout circuit that measures CO_2_ concentration dependent on the thermal time constant of a tungsten hot-wire, where the thermal time constant is a product of the thermal resistance to ambient. The phase shift was detected by the developed delta-sigma modulator and a resolution was extracted of 94 ppm for CO_2_.

Sun et al. [117] integrated two platinum resistors (reference and test) into a portable gas chromatography system. A carrier gas of helium was used and measurement of CO, CH_4_ and some hydrocarbons was shown at a concentration of sub 100 ppm. In this work, finite element analysis was used to optimise the geometry of the two platinum resistors.

Harumoto et al. have developed a thermal gas sensor for detecting hydrogen that employs a temperature sweep on the heated element [118]. The slope of power vs. resistance (and its integral) is then used to calculate the hydrogen concentration. This exercise is carried out on platinum, tungsten and cobalt thin-wires with a discussion on their suitability.

Commercially, Nevada Nano (USA) have created a silicon MEMS based Molecular Property Spectrometer (MPS) capable of measuring multiple gases. Their chip incorporates an array of micro-cantilevers with piezo-electric sensing elements, providing actuation and resonant frequency measurement. All the sensors also have resistive heater that enable temperature modulation and compensation to enhance the sensitivity and also provide cleaning.

#### 6.2.2. 3-Omega Method

Kommandur et al. [27] developed a custom 3-omega conditioning circuit in order to detect the amplitude, phase lag and calculate the in-phase and out-of-phase components, which relates directly to gas concentration using a polysilicon heater. Using this technique, He, Ar, CO_2_ and CH_4_ were measured in hydrogen in concentrations less than 0.5% (5000 ppm). This work also found a characteristic frequency in which the observed phase lag is at its maximum, and thus the highest sensitivity.

Legendre et al. [67] fabricated a nano-scale TCD to be used within a gas chromatography system.

Lofti et al. [119,120,121] have developed a platinum cantilever-based thermal conductivity gas sensor combined with the 3-omega technique. The fabricated sensor successfully detected 60 ppm ammonia in helium. In addition to this, the effect of the gap-size between a heater layer and sensing layer was investigated through a multi-physics simulation and using this optimisation, the detection limit was doubled and the fabricated sensor measured 30 ppm ammonia in helium.

### 6.3. Recent Developments Summary

Table 5 shows a comparison between some of the recent works carried out in the field of thermal conductivity gas sensing. It is hard to directly compare because various gases are investigated that have different thermal conductivity values, thus making the detection limit values not directly comparable.

They can however be useful compared to their prospective application. For example, Carbon dioxide is a major contributor for indoor air pollution and a device would need to be able to measure under ambient room concentration 400 ppm with a resolution of at least 100 ppm, so it can inform the user of unsafe conditions. This is very difficult for thermal conductivity gas sensors due to the similar thermal conductivity values of carbon dioxide and air. An easier, a perhaps more immediate prospect for this technology is hydrogen sensing. This seems especially timely with the interest in a hydrogen economy and the exciting hydrogen fuel cell technology. In these applications leak detection is crucial, which will require a much higher detection limit.

## 7. Conclusions

A study on thermally based gas sensors has been presented and provides a comparison between the different types of thermal conductivity gas sensors. A comprehensive study is also given showing the theory and recent developments of the different methods for extracting gas concentration through the use of thermal resistors. Moreover, current commercial applications that use this technology are discussed and compared with competing technologies.

Currently, it is observed that thermal conductivity gas sensors are not widely used due to two reasons: (i) their lack of discrimination and (ii) their relatively low sensitivity. However, they offer many great advantages including high stability, low-cost, low power consumption, no adsorption/catalyst, fast response time, long lifetime and small footprint. Most recent developments in the literature focus on improving sensitivity, as these are the main barriers that need to be overcome in order to develop a sensing technology that can displace current practices and solidify its place in the market.

This technology can be split into the two distinct approaches of steady-state and transient operation. Whilst steady-state operation offers simplicity, heater modulation enables the measurement of thermal conductivity and thermal diffusivity, effectively opening up a second dimension with which to obtain information.

Thermal conductivity gas sensors are advancing in capability and will be welcomed amongst many applications in the future, especially as part of multi-sensing systems.

## 8. Prospects

### 8.1. Sensitivity

Sensitivity is the main boundary that must be overcome for thermal conductivity gas sensors, in order to see their wide-spread commercial use. Most recent literature is tackling this problem, either by geometric chip design that enhances and optimises the heat transfer from the sensor to the environment, or by improving the read-out techniques and electronics in order to capture small changes in thermal conductivity. With the development of this technology, and the continuing advancement of its sensitivity, it will be able to access current markets with a commercial sensing solution at a fraction of the cost.

The final goal of sensitivity is very dependent on the application in which the sensor will be deployed. For example, a high sensitivity will be required to detect carbon dioxide for indoor air quality whereas it is much easier to detect hydrogen leaks. Thus, with the ever-increasing sensitivity of this technology, more applications will be available.

### 8.2. Selectivity

It is known that most gas sensors face the problems of low selectivity. Sensor responses can be easily influenced by the presence of other gases or mixture of gases as well as varying ambient conditions such as temperature and humidity. There are three ways of dealing with the low sensitivity or enhancing the sensitivity in various applications. The first is within a system where the mixture of gases is known, and a specific gas is to be looked out for. An example includes indoor CO_2_ measurements, where the change in air composition is changed mostly by exhalation. The second way is to modulate the temperature of the heater and take into account the distinctive variation of the thermal conductivity with the temperature of each of the gas and thus being able to separate contributions of different gasses. This requires more complex driving and data processing, but it is entirely plausible. Additionally, the third is to include gas sensors into an array of sensors. Many examples of gas sensor arrays can be seen in electronic nose technology [122], where an array of gas sensors that are selective to different analytes are combined with post-processing techniques such as artificial neural networks or principal component analysis in order to create a system with high selectivity that can detect many different gases.

### 8.3. Manufacturability and Integration

A major barrier towards thermal conductivity gas sensors becoming multi-functional is the integration into large scale systems. Most devices are made using standard CMOS metals and can be manufactured easily in large volumes using commercial foundries. This renders the devices very compatible with integration into electronics, systems-on-chip and systems-in-package.

In addition to this, the design cycle of a sensing product must heavily consider the fabrication and packaging, which can have a strong effect on performance as outlined in this report. The fabrication tolerance can also have an effect on performance and must be stochastically considered for large volume fabrication to ensure that performance stays within the required metrics. An example of such a study for gas sensors can be found here [123].

### 8.4. New Emerging Materials

New materials are an interesting direction that could be explored to access new applications. Gallium Nitride (GaN) offers a low thermal conductivity of 1.3 W/cm·K, meaning thermal losses through the substrate will be reduced, forcing more sensitivity to the environment. In addition to this, GaN are able to operate in much harsher conditions, including higher temperatures, high radiation environments and high pressure. This makes GaN more suitable for applications in aeronautical and harsh planetary environments [124].

There has also been some initial investigation into using nanomaterials, such as Carbon Nano Tubes (CNT) to increase the electro-thermal behaviour of thermal devices. The use of CNTs will increase the surface area in contact with the environment, and could therefore increase the heat transfer between the heated element and the environment, which could ultimately lead to increased sensitivity [104].

Developing new advanced materials requires a thorough understanding of the material and their interfacial properties as well as their behaviour under manufacturing. This needs to be well researched alongside tailoring materials for specific applications in order to investigate, design and develop new materials for micromachined thermal conductivity gas sensors.

### 8.5. Temperature Modulation

Temperature modulation is a key topic and gaining transaction in the commercial world, however it is yet to be widely researched for thermally based gas sensors and is more commonly used for resistive MOX and polymer gas sensors. Selectivity is one of the inherent problems surrounding most gas sensors and temperature modulation is a method of increasing the selectivity by adding an additional parameter which can give information about the environment, without the requirement of detector arrays or additional sensors. A review of semiconductor gas sensor literature pertaining to the use of temperature modulation techniques can is given by Lee and Reedy [125]. Investigation into this area has continued and some more recent examples can be found here [126,127,128].

Interestingly, this technique is yet to be widely researched for gas sensors that are purely thermal. One example is given by Hajjaj et al. whereby an electrothermally heated bridge resonator is used for selective identification of some gases without the need for surface functionalisation of the microstructure, providing longer lifetimes, higher stability and alleviating the bespoke to environment functionalisation process.

Using temperature modulation with thermal-based gas sensors could provide an essential springboard for increasing the selectivity of these devices and should be considered as an emerging approach surrounding this technology.

### 8.6. Artificial Intelligence

Unlike many other sensing scenarios, gas sensing is inherently complex due to the problems it faces, which notably include cross-sensitivity and low selectivity. Applications require gas sensors to measure environments that contain a multitude of constituents, often of which are unknown, as well as being used in varying temperature, humidity and pressure. In order to overcome these challenges, ‘smart’ gas sensing has been proposed recently received considerable attention.

Smart gas sensing is a combination of gas sensor arrays with pattern recognition in order to detect, analyse and differentiate gas mixtures. A recent review on ‘Smart Gas Sensing Technology’ is given by Feng et al. [129] and highlights the emerging attraction of the combination of well-designed sensors and appropriate post-processing techniques.

The future of sensors will be intertwined with ‘smart’ post-processing techniques and Artificial Intelligence (AI), which provide an effective way of increasing the capability and performance of all sensors. Thermal-based gas sensors will also benefit from this combination, which could be employed in several areas and should be an emerging approach of this technology in order to improve its largest problems, sensitivity, selectivity and also long-term drift; and ultimately become a commercially attractive and wide-spread solution.

## Figures and Tables

**Table 1:** A table displaying several gases that are often measured, monitored and controlled in industry with their relevant thermal parameters. The values refer to pressure of 1 bar and temperature of 300 K.

Gas Type	Gas	Thermal Conductivity [10^−3^ W/m·K]	Specific Heat Capacity [J/(K·kg)]	Density [kg/m^3^]
Inert	Air	26.2	1000	1.20
	Ar	17.9	521.5	1.78
	He	156.7	5193.0	0.179
	CO_2_	16.8	852.5	1.84
	Ne	49.8	1030	0.9
	N_2_	26	1041.8	1.126
Toxic	CO	25	1040	1.123
	NH_3_	24.4	2200	0.690
	N_2_O	17.4	880	1.98
	H_2_S	14.6	2240	1.36
Flammable	H_2_	186.9	14310	0.09
	CH_4_	34.1	2235.8	0.657
	C_4_H_10_	16.4	1730	573
	O_2_	26.3	918	1.43
Volatile Organic Compound (VOC)	C_7_H_8_ (Toluene)	134	1130	862
	C_3_H_6_O (Acetone)	11.5	1290	784.5
	C_2_H_6_O (Ethanol)	14.4	1600	785.3
	CH_2_O (Formaldehyde)	-	-	815
	C_8_H_10_ (Xylene)	-	1720	860
	C_6_H_6_ (Benzene)	-	1800	876

**Table 2:** A table summarising advantages and disadvantages of the three main methods for temperature transduction.

Transduction Principle	Thermo-Resistive	Thermo-Electric	Thermo-Electronic
Example sensor	RTD Thermistor	Thermocouple Thermopile	Diode Transistor
Advantages	Cheap Good linearity High stability Can be suspended	Self-powered Wide temperature range Simple Accurate Fast response time	Linear Wide temperature range Low current No self-heating Small
Drawbacks	Current sourced Self-heating Electro-migration	Non-linear Low voltage Low stability Cold junction required	High thermal noise Large occupied area High resistance

**Table 3:** A table summarising some typical characteristics for the most common GC detectors that are used in applications and industry.

Detector	Selectivity	LoD	Cost
Thermal Conductivity Detector (TCD)	Responds if thermal conductivity of gas is different to that of the carrier gas.	1 ng/mL	Low
Flame Ionisation Detector (FID)	Organic compounds.	1 pg(C)/s	Medium
Electron-Capture Detector (ECD)	Electron capturing compounds, such as halogens.	10 fg/s	High
Electrolytic Conductivity Sensor (ELCD)	Halogens and S.	1 pg CI/s	Medium
Atomic Emission Detector (AED)	Element selective.	0.1–50 pg/s	High

**Table 4:** Comparison between the materials that are commonly used for the metal layer within CMOS MEMS sensors (as part of the CMOS sequence or post-CMOS), and thus used frequently for the heated element.

Property (Units)	Al	W	Au	Pt
Electrical Conductivity (S/m)	377	183	488	94
Melting Point (°C)	660	3410	1064	1772
Density (kg/m^3^)	2720	19,350	19,320	21,450
TCR (10^−4^/°C)	39	45	34	30
Thermal Conductivity (W/m/K)	236	177	319	72
Specific Heat Capacity (J/K/kg)	904	134	129	133
Linear Expansivity (K^−1^)	23.1	4.5	14.2	8.8
Young’s Modulus (GPa)	70	411	78	168
Yield Strength (MPa)	50	750	200	<14
Poisson’s Ratio	0.35	0.28	0.44	0.38

**Table 5:** A comparative table between some of the recent works in thermal conductivity gas sensing.

Reference	Operation	Measured Gas	Heater Material	Limit of Detection	Comments
[46]	Steady-state	Hydrogen	Platinum	2000 ppm	Helium used in experiment
[105]	Steady-state	Helium	Carbon and gold	142 ppm	Low power consumption of 240 nW using PWM
[110]	Steady-state	Carbon dioxide	Tungsten	200 ppm	Ratiometric readout circuit
[4]	Steady-state	Tungsten	Tungsten	100 ppm	Differential measurement on single membrane
[63]	Transient	Tungsten	Polysilicon	178 ppm	Data obtained for other gases as well
[102]	Transient	Polysilicon	Polysilicon	3313 ppm	Data obtained for other gases as well
[115]	Transient	Polysilicon	Tungsten	94 ppm	Uses thermal time constants
[116]	Transient	Tungsten	Platinum	5 ppm	Other gases investigated, GC system.
[117]	Transient	Platinum, tungsten and cobalt	Platinum, tungsten and cobalt	10,000 ppm	Uses a temperature modulation method
[119]	Transient	Platinum	Platinum	30 ppm	Cantilever based design.
